# *OsRAMOSA2* Shapes Panicle Architecture through Regulating Pedicel Length

**DOI:** 10.3389/fpls.2017.01538

**Published:** 2017-09-12

**Authors:** Huan Lu, Zhengyan Dai, Ling Li, Jiang Wang, Xuexia Miao, Zhenying Shi

**Affiliations:** ^1^National Key Laboratory of Plant Molecular Genetics, Institute of Plant Physiology and Ecology, Shanghai Institutes for Biological Sciences, Chinese Academy of Sciences Shanghai, China; ^2^University of Chinese Academy of Sciences Shanghai, China; ^3^Key Laboratory of Insect Developmental and Evolutionary Biology, Institute of Plant Physiology and Ecology, Shanghai Institutes for Biological Sciences, Chinese Academy of Sciences Shanghai, China; ^4^Ministry of Agriculture Key Laboratory of Urban Agriculture (South), Plant Biotechnology Research Center, School of Agriculture and Biology, Shanghai Jiao Tong University Shanghai, China

**Keywords:** LBD protein, *RA2* gene, panicle architecture, pedicel, transcriptional factors

## Abstract

The panicle architecture of rice is an important characteristic that influences reproductive success and yield. It is largely determined by the number and length of the primary and secondary branches. The number of panicle branches is defined by the inflorescence meristem state between determinacy and indeterminacy; for example, the maize *ramosa2* (*ra2*) mutant has more branches in its tassel through loss of spikelet determinacy. Some genes and factors influencing the number of primary and secondary branches have been studied, but little is known about the molecular mechanism underlying pedicel development, which also influences panicle architecture. We report here that rice *OsRAMOSA2* (*OsRA2*) gene modifies panicle architecture through regulating pedicel length. Ectopic expression of *OsRA2* resulted in a shortened pedicel while inhibition of *OsRA2* through RNA interference produced elongated pedicel. In addition, *OsRA2* influenced seed morphology. The OsRA2 protein localized to the nucleus and showed transcriptional activation in yeast; in accordance with its function in pedicel development, *OsRA2* mRNA was enriched in the anlagen of axillary meristems, such as primary and secondary branch meristems and the spikelet meristems of young panicles. This indicates a conserved role of *OsRA2* for shaping the initial steps of inflorescence architecture. Genetic analysis revealed that *OsRA2* may control panicle architecture using the same pathway as that of the axillary meristem gene *LAX1* (*LAX PANICLE1*). Moreover, *OsRA2* acted downstream of *RCN2* in regulating pedicel and branch lengths, but upstream of *RCN2* for control of the number of secondary branches, indicating that branch number and length development in the panicle were respectively regulated using parallel pathway. Functional conservation between *OsRA2* and *AtLOB*, and the conservation and diversification of *RA2* in maize and rice are also discussed.

## Introduction

Rice (*Oryza* *sativa* L.) provides a staple food for more than half of the world’s population and a model plant for the molecular study of cereal crops. Panicle architecture is one of the main features influencing reproductive success and contributes directly to grain yield (Sakamoto and Matsuoka, 2008; Li et al., 2011), so has been a major target of cereal crop domestication.

The panicle architecture of cereal plants is mainly determined by the number and length of primary branches (PBs) and secondary branches (SBs). These are largely established by iterations of branching that are governed by spatiotemporal developmental decisions in inflorescence, branch and spikelet meristems between the maintenance of determinacy and indeterminacy (Kellogg et al., 2013; Teo et al., 2014; Zhang and Yuan, 2014). *Arabidopsis* genes *LEAFY* (*LFY*), *APETALA1* (*AP1*), *CAULIFLOWER* (*CAL*), *FRUITFULL* (*FUL*) and *Antirrhinum* genes *FLORICAULA* (*FLO*) and *SQUAMOSA* (*SQUA*) promote the formation of terminal floral and determinacy (Coen et al., 1990; Huijser et al., 1992; Mandel and Yanofsky, 1995; Weigel and Nilsson, 1995; Ferrandiz et al., 2000), while *TERMINAL FLOWER1* (*TFL1*) in *Arabidopsis* and *CENTRORADIALIS* (*CEN*) in *Antirrhinum* promote an indeterminate inflorescence (Bradley et al., 1996, 1997). Accordingly, *RCN1* and *RCN2*, rice homologs of *TFL*/*CEN*, lead to an indeterminate inflorescence and a more branched panicle when ectopically expressed (Nakagawa et al., 2002). These indeterminacy promoting and inhibiting factors also interact with each other, for instance, CEN interacts with FLO in regulating inflorescence architecture (Bradley et al., 1996, 1997). However, it is not known if *OsRA2* interact with other genes in panicle development?

The maize *ra2* mutant has an increased number of tassel branches through loss of spikelet determinacy (Bortiri et al., 2006). The RA2 protein, a member of the Lateral Organ Boundaries Domain (LBD) protein family, is a specific regulator of plant organ development, and has been shown to function not only in various developmental processes in the leaf, root, inflorescence, microspore, and pollen, but also in plant regeneration, photomorphogenesis, and plant defense (Shuai et al., 2002; Xu et al., 2016). Barley *HvRA2* plays conserved role in determining spikelet determinacy and controlling lateral spikelet fertility (Koppolu et al., 2013).

Branch meristems in the panicle are axillary meristems (AMs) that determine the complexity of the whole plant architecture (Tabuchi et al., 2011). *LAX1* is a key regulator of AM initiation and maintenance in rice (Komatsu K. et al., 2003; Oikawa and Kyozuka, 2009), *MONOCULM1* (*MOC1*) regulates AM formation and when mutated leads to fewer branches in the panicle (Li et al., 2003), while LAX2 is expressed in all the AMs that give rise to PBs, SBs and spikelets, and physically interacts with LAX1, the *lax1lax2* double mutant develops almost no branches in the panicle (Tabuchi et al., 2011). *FRIZZY* *PANICLE* (*FZP*) restrains overgrowth of AM and results in excessive ramification of rachis-branches when mutated (Komatsu et al., 2001; Komatsu M. et al., 2003). *MOC1* and *LAX1*, and some microRNAs (miRNAs) are involved in the consistent development of panicle architecture and tillering (Wang L. et al., 2015), but mostly, panicle development is distinctly regulated (Liang et al., 2014). It is not known if any genetic crosstalk exists between AM development and meristem determinacy during panicle development.

Phytohormones are also thought to influence panicle architecture (Mcsteen, 2009), with up-regulation of *OsCKX2* (*CYTOKININ OXIDASE*) in the cytokinin pathway resulting in an increased number of PBs and SBs (Ashikari et al., 2005; Han et al., 2014). Moreover, post-transcriptional regulation by miRNAs and their targets was found to regulate panicle branching (Wang L. et al., 2015), as demonstrated by the loss of function of maize miR172 which caused a loss of spikelet determinacy and an excessive number of branches (Chuck et al., 2007). *OsSPL14* negatively modulates the transition to spikelet meristem (Rhoades et al., 2002; Jiao et al., 2010; Miura et al., 2010; Luo et al., 2012; Wang et al., 2017).

The pedicel length is another key contributor to the diversity of inflorescence architecture. Although a few genes and factors regulating the number of PBs and SBs have been identified, little is known about the regulation of their length. *SP1* was reported to negatively regulate the panicle length (Li et al., 2009). And even less is known about the control of pedicel development, with only two associated genes in *Arabidopsis* and one in tomato reported (Yamaguchi and Komeda, 2013; Wang D. et al., 2015).

Because of the complexity of panicle constitution and development, its molecular mechanism and regulation network is poorly understood. Therefore, the present study investigated the function of *OsRA2* in rice. *OsRA2* was found to regulate seed morphology and pedicel development in the panicle, and its expression profile corresponded well with its function in these aspects. Preliminary genetic analysis revealed that *OsRA2* might control rice panicle architecture using the same pathway as that of *LAX1*. Moreover, *OsRA2* acted downstream of *RCN2* in regulating pedicel and branch lengths, but upstream of *RCN2* for control of the number of SBs, indicating that branch number and length development in the panicle were respectively regulated using parallel pathway.

## Materials and Methods

All experimental protocols and plant materials were approved by Shanghai Institutes for Biological Science, Chinese Academy of Sciences.

### Plant Materials

Wild type rice ZH11 (*Oryza* *sativa* L. subsp. *japonica* cv. Zhonghua No.11), the *lax1*, A989 mutants, and all the other transgenic plants used in this study were grown in a paddy field or in pots in a greenhouse under standard growth conditions.

### Phylogenetic Analysis

Homolog sequences of RA2 from *Schizachyrium* *sanguineum* (SsRA2), *Sorghum* *bicolor* (SbRA2), *Andropogon* *hallii* (AhRA2), *Cymbopogon* *flexuosus* (CfRA2), *Andropterum* *stolzii* (AsRA2), *Phacelurus digitatus* (PdRA2), *Zea* *mays* (ZmRA2), *Chrysopogon* *gryllus* (CgRA2), *Hordeum* *vulgare* (HvRA2), *Oryza* *sativa* (OsRA2), *Loudetia* *sp*. *MCE* (LsRA2), AtASL4 and AtLBD25 were obtained from Uniprot^1^ (Supplementary Table S1), homologous sequences of AtASL4 and AtLBD25 were obtained from the National Centre for Biotechnology Information^2^. Multiple sequence alignments of proteins were conducted using ClustalX Version 1.83 (Thompson et al., 1997). A phylogenetic tree of the sequences was reconstructed by the neighbor-joining (NJ) method by MEGA3 (Kumar et al., 2004).

### Plasmids and Constructs

To generate *OsRA2* RNAi plasmid pdsRNAiOsRA2, a gene-specific fragment was amplified by KOD-plus DNA polymerase (*TOYOBO*) using primers *OsRA2*-RNAiF/*OsRA2*-RNAiR and cloned into pCAMBIA1301RNAi vector. To obtain the pUbi::OsRA2 plasmid, full-length cDNA of *OsRA2* was amplified using primers *OsRA2*-*Bam*HIA/*OsRA2*-*Kpn*I and cloned into pCAMBIA1301UbiNos vector using *Bam*HI and *Kpn*I.

### Genetic Transformation of Rice

The embryogenic calli induced from immature ZH11 seeds were transformed by *Agrobacterium tumefaciens* infection and transgenic plants were regenerated (Hiei et al., 1994). Primer pairs Ubi 90+/*OsRA2*-*Kpn*I and ocs-160/*OsRA2*-RNAiF were used to check pUbi::OsRA2 and pdsRNAiOsRA2 transgenic plants respectively.

### Quantitative Real-Time (qRT-PCR) PCR Assays

For qRT-PCR assays, total RNAs were extracted from various tissues, panicles and seeds at different developmental stages using TRIzol reagent (*Invitrogen*), and reverse transcribed using oligo(dT) primer and ReverAce (*TOYOBO*). cDNA was synthesized from 2 μg of total RNA treated with DnaseI (*TOYOBO*) and used as templates for RT-PCR or qRT-PCR. Rice *actin* gene was used for normalization. Single and double asterisks represent significant difference determined by the Student’s *t*-test at ^∗^*P* < 0.05 and ^∗∗^*P* < 0.01 respectively.

For *OsRA2* expression analysis, the roots, leaves, sheaths, culms, shoot apical meristem (SAM) were collected from around 30-day-old plants; panicles meristems less than 1 cm were collected under microscope, and flowers were collected from heading stage.

### *In* *Situ* Hybridization Analysis

*In* *situ* hybridization was performed according to previous description (Coen et al., 1990). Young panicles at different developmental stages were fixed in 4% paraformaldehyde PBS solution overnight at 4°C, dehydrated through a concentration grade of ethanol, cleared through a dimethylbenzene series, then infiltrated through a series of paraffin (*Sigma–Aldrich*) melted at 60°C, and finally embedded in 100% paraffin. The longitudinal sections of young panicle were sectioned into 7 μm strips and mounted on RNase-free glass slides (Sigma). Gene-specific fragments of *OsRA2* and *OSH1* were amplified using primer pairs *OsRA2*-*insitu* F/*OsRA2*-*insitu* R and *OSH1*F/*OSH1*R respectively, and cloned into pBSK(-) vector, then linearized and used as templates for digoxigenin-labeled sense and antisense RNA probes, which were transcribed *in vitro* using a DIG RNA labeling kit (*Promega*).

### Measurement of Grain Traits

Fifty grains from each sample were used for measurement of grain traits, including grain length (GL), grain width (GW) and grain thickness (GT). Length-to-width ratio (LWR) was obtained by dividing GL by GW. The 1,000-grain weight was got by measuring 100 grains in 10 biological repeats. Data was shown as mean ± SD.

### Anatomical Analysis

Pedicels of ZH11, dsRNAiOsRA2 and pUbi::OsRA2 plants were cut transversely and fixed in 50% FAA at 4°C overnight after vacuuming. After dehydration in several concentrations of ethanol, samples were embedded in epoxide resin and cut into 2–3 μm slices and spread at 42°C on a hot platform overnight, stained using 0.5% toluidine Blue O and sealed for observation under the microscope (Wang et al., 2010). For the thickness measurement, at least 10 pedicels were made into histological sections and measured under microscope.

### *Trans*-Activation Assay

Full-length cDNA of *OsRA2* was amplified and cloned in-frame with the LexA DNA-binding domain using primers Osa_yRA2EcoRI and Osa_ yRA2BamHI in PEG202 (His3, 2 μm, Amp^r^, ADH constitutive promoter, LexA DNA-binding domain) (DupLEX-A^TM^ system) (*OriGene Technologies*). The pEG202-OsRA2 and pEG202 plasmids were introduced into yeast strain EGY48 (*MATtrp1his3ura3leu2*::*6lexAop*-*LEU2*) harboring the reporter plasmid pSH18-34 (URA3, 2 mm, Amp^r^, LexAops-*lacZ*). Yeast clones were grown on SD medium without histidine or uracil in the presence of X-gal (5-bromo-4-chloro-3-indolyl-β-galactopyranoside) for 2 days at 30°C.

### Sub-cellular Localization of OsRA2

Full length cDNA of *OsRA2* was cloned in frame with GFP into p1301-GFP using primers *OsRA2*-*Bam*HIB/*OsRA2*-*Pst*I and genetically transformed into ZH11. Root apices from the transgenic plants were observed through a confocal laser scanning microscopy (OLYMPUS FV1000).

Primer sequences used in this study were listed in Supplementary Table S2.

## Results

### Identification of *OsRA2* Gene As Homologous to Maize *RA2*

**FIGURE 1:**
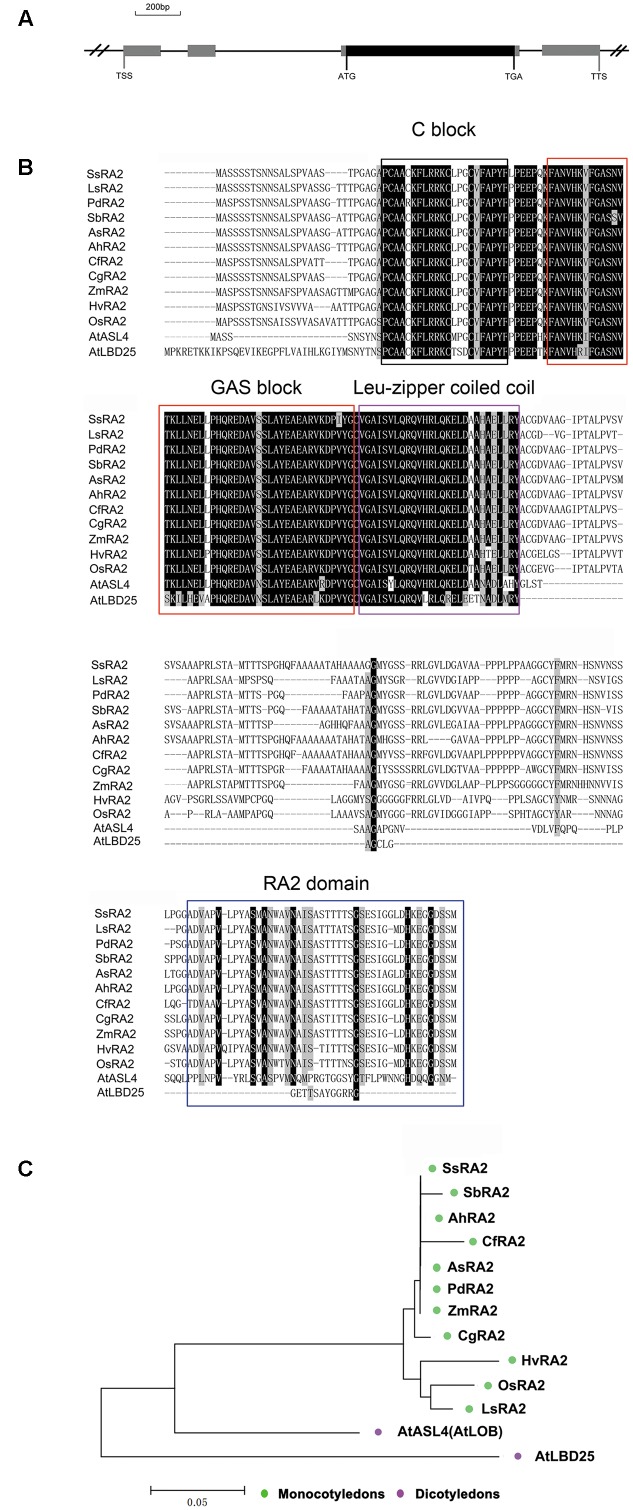
Analysis of *OsRA2* and comparison with other grass RA2 proteins and *Arabidopsis* LBD proteins. **(A)** Schematic of *OsRA2*. Black box represents the protein-coding region and gray ones represent exons. **(B)** Alignment of the deduced full-length amino acid sequences of SsRA2, SbRA2, AhRA2, CfRA2, AsRA2, PdRA2, ZmRA2, CgRA2, HvRA2, OsRA2, LsRA2, AtASL4 and AtLBD25. The respective species are listed in the Materials and methods. Protein domains are framed in black for C-block, red for GAS-block, purple for Leu-zipper coiled coil and blue for the RA2 domain. **(C)** Phylogenetic tree of grass RA2 proteins and *Arabidopsis* LBD proteins.

A search of the public databases^3^^,^^4^^,^^5^ revealed that only one gene, *OsRA2*, of the rice genome was homologues to maize *RA2*. *OsRA2* contains four exons and three introns, with an open reading frame (ORF) in the third exon encoding a protein of 251 amino acids (**Figure 1A**). The deduced OsRA2 protein is composed of a characteristic N-terminal LOB domain and a variable C-terminal RA2 domain. The LOB domain is characteristic of the LBD family proteins (Bortiri et al., 2006), and in OsRA2 consists of a C block that functions in DNA-binding, a leucine zipper coiled coil motif for protein dimerization, and a Gly-Ala-Ser block (**Figure 1B**). The C block is represented by a CX_2_CX_6_CX_3_C motif containing four conserved cysteine (C) residues and other non-conserved residues (X), and the leucine-zipper-like motif includes five hydrophobic amino acids separated by six variable amino acid residues (Shuai et al., 2002; Matsumura et al., 2009; Majer and Hochholdinger, 2011). Phylogenetic alignment of protein sequences revealed that RA2 is highly conserved among monocotyledons, while some diversification has occurred between grass RA2 proteins and *Arabidopsis* LBD proteins (**Figures 1B,C**).

### *OsRA2* Regulated Pedicel Development and Other Panicle Characteristics in Rice

To investigate the function of *OsRA2*, we carried out genetic analysis, using gene-specific doubled–stranded RNA interference (dsRNAi) and ectopic expression technologies.

**FIGURE 2:**
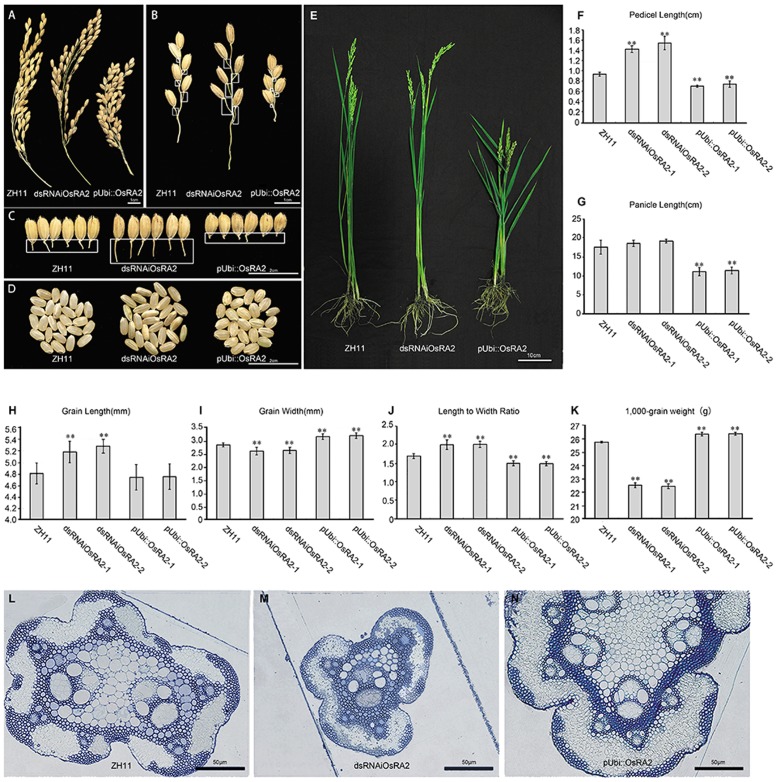
Phenotypes of *OsRA2* transgenic plants. **(A–C)** Phenotypes of ZH11, dsRNAiOsRA2 and pUbi::OsRA2 plants with respect to panicles, PBs and pedicels, respectively. White rectangles in **(B)** indicate the position of pedicels in **(C)**. **(D)** The seed phenotype of ZH11, dsRNAiOsRA2 and pUbi::OsRA2 plants. **(E)** Morphology of ZH11, dsRNAiOsRA2 and pUbi::OsRA2 plants. **(F,G)** Statistical analysis of pedicel and panicle length among ZH11, dsRNAiOsRA2 and pUbi::OsRA2 plants. **(H–K)** Statistical analysis of grain traits among ZH11, dsRNAiOsRA2 and pUbi::OsRA2 plants. Values in **(F–K)** are means ± SE. *n* = 30 panicles in **(F–K)** and 10 replicates in **(K)**. **(L–N)** Cross sections of the pedicel of ZH11, dsRNAiOsRA2 and pUbi::OsRA2 plants. Student’s *t*-test at ^∗^*P* < 0.05 and ^∗∗^*P* < 0.01.

We obtained a total of 27 T0 generation plants from the dsRNAiOsRA2 transformation, of which 24 showed elongated pedicels (**Figures 2A–C**). Successful down-regulation of *OsRA2* in these lines was verified (Supplementary Figure S1). The pedicels of the panicles in dsRNAiOsRA2 plants were longer than those in wild type (WT) ZH11 (>1.4 cm vs. 1 cm; **Figures 2B,C,F**), but there was little change in the number and length of the PBs and SBs (Supplementary Figures S2A–D).

By contrast, pUbi::OsRA2 plants in which OsRA2 was over expressed showed a compact panicle and shortened pedicel (**Figure 2A** and Supplementary Figure S1), with an average pedicel length of 0.8 cm (**Figure 2F**), which was obviously shorter than WT (**Figures 2B,C,F**). Furthermore, the PBs, SBs, and the entire panicle were shortened (**Figure 2G** and Supplementary Figures S2A,B). There was little change in the number of the PBs (Supplementary Figure S2C), but the number of SBs decreased (Supplementary Figure S2D), such that the entire panicle was shortened and condensed (**Figure 2A**).

Based on the phenotype of dsRNAiOsRA2 and pUbi::OsRA2 transgenic plants, *OsRA2* appears to regulate the pedicel length in rice.

### *OsRA2* Influenced the Longitudinal Elongation of the Entire Plant

Besides controlling pedicel length, *OsRA2* also regulated the thickness of the pedicel, which was decreased in dsRNAiOsRA2 plants and increased in pUbi::OsRA2 plants (**Figure 2C** and Supplementary Figure S3); the thicknesses of the branches and the culms in their panicles were altered accordingly (**Figures 2A,B**). We further examined the transverse structure of the pedicels, and identified fewer vascular bundles in dsRNAiOsRA2 plants (**Figure 2M**), and more in pUbi::OsRA2 plants (**Figure 2N**), as compared with ZH11 (**Figure 2L**). So that, dsRNAiOsRA2 plants had thinner pedicels, while pUbi::OsRA2 plants had thicker ones.

*OsRA2* also influenced the shape of the grains in transgenic plants. In dsRNAiOsRA2 plants, the grains were elongated (**Figures 2D,H**), but less wide (**Figure 2I**), so that the GL to width ratio was increased (**Figure 2J**). In pUbi::OsRA2 plants, the GL did not change (**Figure 2H**), but the GW increased (**Figure 2I**), so that the length to width ratio was decreased (**Figure 2J**). As a result, the 1,000-grain weight of dsRNAiOsRA2 plants decreased, while that of pUbi::OsRA2 plants increased (**Figure 2K**).

At the whole plant level, the height of pUbi::OsRA2 plants decreased, while that of dsRNAiOsRA2 plants remained unchanged (**Figure 2E** and Supplementary Figure S2E).

Taken together, *OsRA2* appears to regulate pedicel development and seed development, and its over-expression also influences plant height in rice.

### The *OsRA2* Expression Pattern Conformed to Its Function in Panicle Development

**FIGURE 3:**
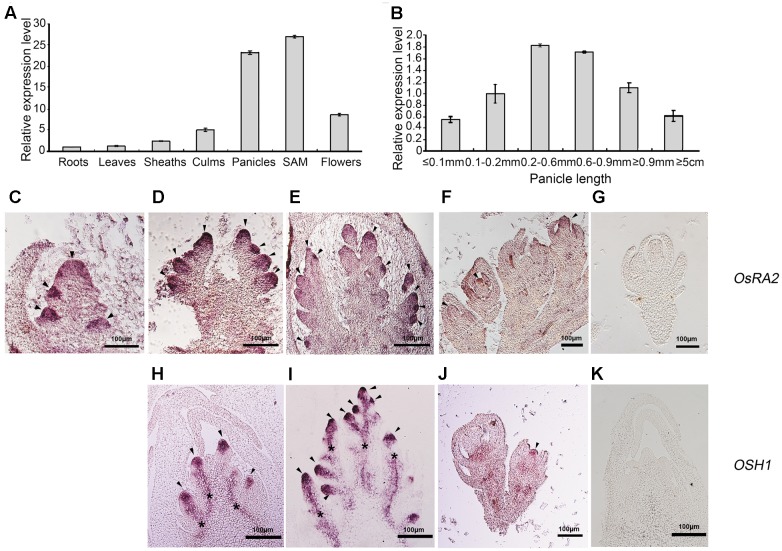
Expression profile of *OsRA2.* **(A)** *OsRA2* expression in various plant organs. **(B)** *OsRA2* expression in various stages of panicle development. **(C–G)** mRNA *in* *situ* hybridization of *OsRA2.* **(C)** *OsRA2* expression in the primordia of the PBs (arrowheads). **(D)** *OsRA2* expression in the primordia of the SBs (arrowheads). **(E)** *OsRA2* expression in the primordia of the spikelet meristem (arrowheads). **(F)** *OsRA2* expression in the developed spikelets (arrowhead). **(G)** Sense probe of *OsRA2* hybridization. **(H–K)** mRNA *in* *situ* hybridization of *OSH1*. **(H)** *OSH1* expression in the primordia of the PBs (arrowheads). **(I)** *OSH1* expression in the primordia of the SBs (arrowheads). **(J)** *OsRA2* expression in the developed spikelets (arrowheads). **(K)** Sense probe of *OSH1* hybridization.

Gene expression is tightly linked to its function. To detect *OsRA2* expression, we carried out quantitative real-time RT-PCR (qRT-PCR), semi-quantitative RT-PCR and *in* *situ* hybridization analyses. *OsRA2* was shown to mainly express in young panicles and in shoot apical meristems (SAMs) at the vegetative stage. Weaker expression was also detected in the culms and flowers, and basal expression was revealed in the roots, leaves, and sheaths (**Figure 3A**). Based on our observation that *OsRA2* regulates pedicel development, and that *OsRA2* over-expression results in shortened PBs, SBs, and panicles, we next examined the expression of *OsRA2* at different stages of panicle development (indicated by panicle length). Expression of *OsRA2* gradually increased and then decreased during panicle development, with the highest transcription level observed in 0.2–0.6 mm and 0.6–0.9 mm panicle meristems (**Figure 3B**). The 0.2–0.6 mm panicle meristem corresponds to the stage of PBs formation and development, while the 0.6–0.9 mm panicle meristem corresponds to the formation and development of SBs, tertiary branches and pedicels. This suggests that levels of *OsRA2* expression are in accordance with its function in pedicel development and panicle development.

We further used *in* *situ* hybridization to locate *OsRA2* mRNA in the young panicles. *OsRA2* mRNA was highly enriched in the anlagen of PB meristems (**Figure 3C**), SB meristems (**Figure 3D**), spikelet meristems (**Figure 3E**), and floral organ meristems (**Figure 3F**) as compared with the sense probe (**Figure 3G**). *ORYZA* *SATIVA* *HOMEOBOX1* (*OSH1*), an ortholog of *KNOTTED1* (*KN1*) in rice, is highly expressed in meristematic cells and is required for meristem maintenance (Sentoku et al., 1999; Oikawa and Kyozuka, 2009; Tsuda et al., 2011). Similarly, *OSH1* transcripts were detected in the anlagen of PB meristems (**Figure 3H**), SB meristems (**Figure 3I**), and floral organ meristems (**Figure 3J**), as compared with the sense probe (**Figure 3K**), indicating the meristematic character of the region enriched in *OsRA2* mRNA transcripts.

### Characterization of the OsRA2 Protein

Most LBD proteins are transcription factors (Husbands et al., 2007). OsRA2 was shown to have a putative nuclear localization signal in its N-terminal LOB domain (**Figure 1B**)^6^, indicating that it is also likely to be a transcription factor.

**FIGURE 4:**
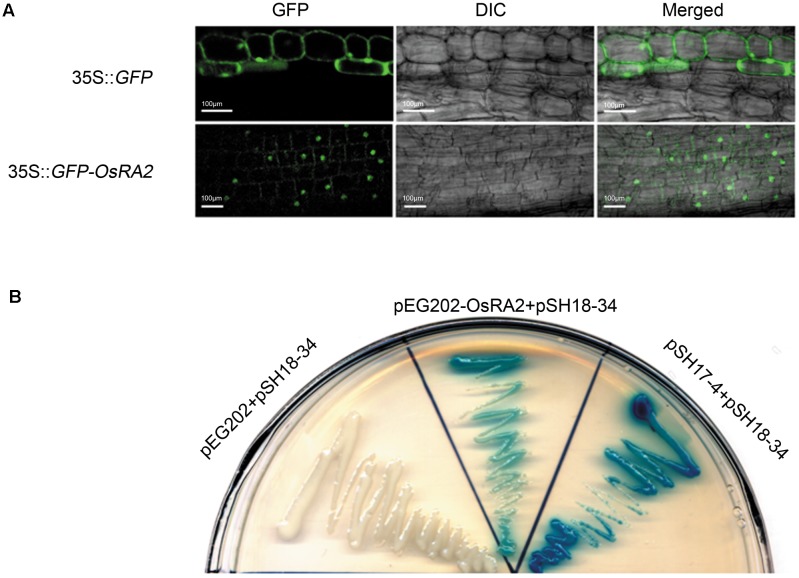
Characterization of the OsRA2 protein. **(A)** Sub-cellular localization of the GFP signal in the 35S::GFP-OsRA2 and the 35S::GFP transgenic plants. **(B)** OsRA2 showing trans-activating activity in the yeast two-hybrid system. Yeast cells transformed with OsRA2 and reporter pSH18-34 turned blue similar to the positive controls pSH17-4 and pSH18-34.

To study the cellular localization of OsRA2, we constructed transgenic plants containing OsRA2 fused in-frame to Green Fluorescent Protein (GFP) at the C-terminal. The fluorescence signal from the fusion protein was observed in the nuclei of p35S::GFP-OsRA2 root tips (**Figure 4A**); but was ubiquitously distributed in the cytoplasm of control plants carrying an empty GFP vector (p35S::GFP) (**Figure 4A**), suggesting that OsRA2 is a nuclear-localized protein. Examination of the phenotype of transgenic plants suggested that OsRA2 functions normally when fused with GFP, because p35S::GFP-OsRA2 plants showed a dwarf phenotype similar to pUbi::OsRA2 plants (Supplementary Figure S4A), and the panicles of p35S::GFP-OsRA2 plants were shortened (Supplementary Figure S4B), similar to pUbi::OsRA2 plants.

We next detected if OsRA2 contained transcriptional activity. We used two plasmids (effector pEG202 and reporter pSH18-34) selected from a DupLEX-A^TM^ yeast two-hybrid system (Zhu et al., 2003). Full-length *OsRA2* cDNA was fused in-frame with LexA protein and transformed into recipient yeast containing *LacZ*, whose promoter elements can be recognized by LexA. Yeast clones transformed with OsRA2-lexA showed a blue color similar to the positive control when supplemented with the β-galactosidase substrate X-gal, indicating that the OsRA2 protein has trans-activation activity (**Figure 4B**).

Together, these experiments strongly indicate that OsRA2 protein is a transcription factor.

### Possible Cross-Talk between *OsRA2* and Other Panicle Development Related Genes

**FIGURE 5:**
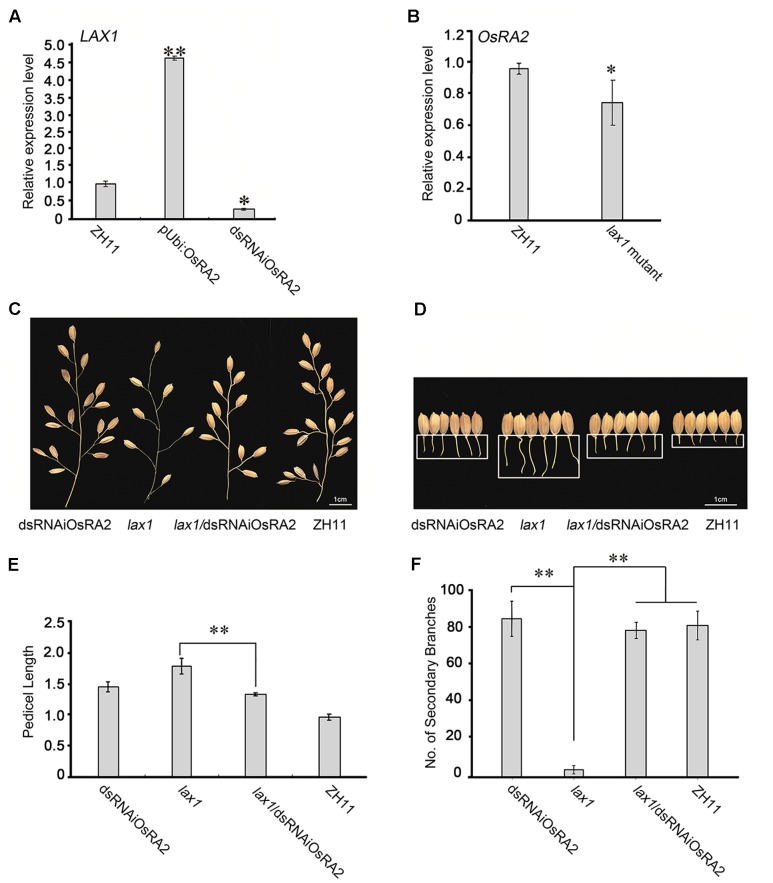
Possible relationships between *OsRA2* and other genes. **(A)** qRT-PCR analyses of *LAX1* in the ∼2 cm young panicles of pUbi::OsRA2 and dsRNAiOsRA2 plants. **(B)** qRT-PCR analyses of *OsRA2* in the *lax1* mutant. **(C,D)** PBs and pedicels of the dsRNAiOsRA2 plant, *lax1* mutant, *lax1*/dsRNAiOsRA2 plant, and ZH11. **(E,F)** Statistical analysis of the pedicel length and number of SBs in dsRNAiOsRA2 plants, *lax1* mutants, *lax1*/dsRNAiOsRA2 plants and ZH11. Values are means ± SE *n* = 15 panicles. Single and double asterisks represent significant difference determined by the Student’s *t*-test at ^∗^*P* < 0.05 and ^∗∗^*P* < 0.01 respectively.

To explore the pathway by which *OsRA2* regulates pedicel development, we detected the expression of panicle-regulating genes in dsRNAiOsRA2 and pUbi::OsRA2 plants (Supplementary Figure S5). Among them, *LAX1*, *MOC1* and the *RCNs* are involved in meristem initiation and development (Nakagawa et al., 2002; Komatsu K. et al., 2003; Li et al., 2003), *SP1* regulates the panicle length (Li et al., 2009), *DEP1* regulates number of branches and panicle length (Huang et al., 2009), and *OSH1* is a meristem marker gene (Tsuda et al., 2011). *LAX1* was shown to be up-regulated in pUbi::OsRA2 plants but down-regulated in dsRNAiOsRA2 plants (**Figure 5A**). *lax1* mutants contain longer pedicel because of defects in initiation or maintenance of the lateral and terminal spikelets (Komatsu K. et al., 2003; Tabuchi et al., 2011), so we wondered if *LAX1* is transcriptionally regulated by *OsRA2.* However,we found no OsRA2 binding motifs in the *LAX1* promoter^7^. We also performed a yeast two-hybrid assay to check an interaction between OsRA2 and LAX1, but negative result turned out. Therefore, it remains unclear if any direct regulation or interaction between *OsRA2* and *LAX1*exists.

*OsRA2* expression was down-regulated in the *lax1* mutant (**Figure 5B**), so to investigate the genetic relationship between *OsRA2* and *LAX1*, we crossed the *lax1* mutant with dsRNAiOsRA2 plants. And the number of SBs in the hybrid increased as compared with that in the *lax1* mutant (**Figures 5C,F**), suggesting that *LAX1* functions upstream of *OsRA2* in determining the number of SBs. F2 *lax1*/dsRNAiOsRA2 plants showed longer pedicels compared with WT, which were more similar to those of dsRNAiOsRA2 plants (**Figures 5D,E**). Thus, *LAX1* might also function upstream of *OsRA2* in regulating pedicel length.

**FIGURE 6:**
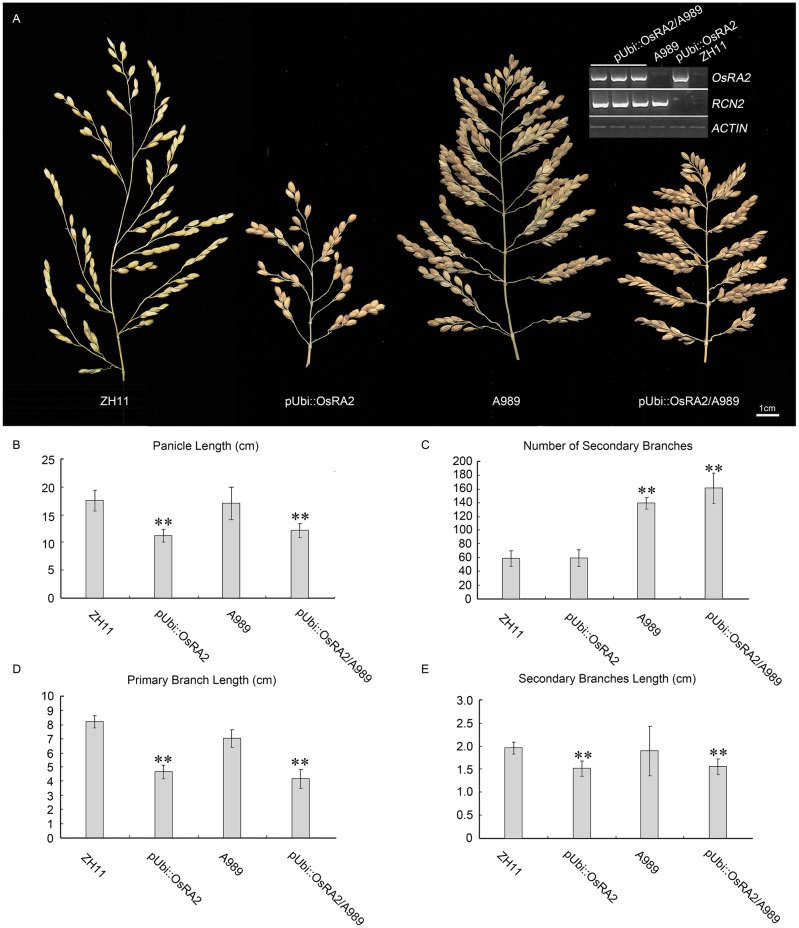
Phenotypic analysis of pUbi::OsRA2 plants and the A989 mutant. **(A)** Panicle characteristic of the pUbi::OsRA2 plant and the A989 mutant and their hybrid. Expression levels of *RCN2* and *OsRA2* in the hybrids are shown in the gel image (top right, inset). **(B–E)** Respective panicle characters of the pUbi::OsRA2 plant, A989 mutant and their hybrid. Student’s *t*-test at ^∗∗^*P* < 0.01.

The over expression of *RCN2* showed indeterminacy with increased PBs and SBs (Ikeda-Kawakatsu et al., 2012). To study the possible genetic relation between *RCN2* and *OsRA2*, we crossed the pUbi::OsRA2 plants to the T-DNA insertion mutant of *RCN2* in our lab (A989, **Figure 6A**), in the F2 generation, the characteristics of pUbi::OsRA2 plants and those of A989 were effectively additive, with the hybrid showing the shortened branches and panicles similar to those of pUbi::OsRA2 plants (**Figures 6A,B,D,E**), and an increased number of SBs similar to A989 (**Figures 6A,C**). This indicates that *OsRA2* acts downstream of *RCN2* in terms of pedicel and branch lengths, but upstream of *RCN2* in terms of the number of SBs.

## Discussion

*OsRA2* encodes a plant specific LBD protein. Previous studies have suggested that the function and expression pattern of *RA2* genes are evolutionarily conserved among grasses (Bortiri et al., 2006). *OsRA2* mRNA can be detected from the P3 stage (corresponding to the stage of PB meristems) and in the anlagen of PB meristems and spikelet meristems (Bortiri et al., 2006; Oikawa and Kyozuka, 2009). In this study, we provide further evidence that the sequences of the RA2 protein are highly conserved among several monocotyledons and dicotyledons (**Figures 1B,C**). We also demonstrate that *OsRA2* has a novel expression profile and function in seed morphology compared with *RA2* in maize. *OsRA2* mRNA expression is highly enriched in AMs such as PB and SB meristems and spikelet meristems, which is in accordance with its function in regulating pedicel length and the shortened PBs and SBs in pUbi::OsRA2 plants. *OsRA2* is also expressed in the floral organ meristem (**Figure 3F**), which is in accordance with its function in seed morphology.

Protein sequence conservation among species implies a conserved function. Many LBD proteins are characterized by their expression in the boundary between SAM and lateral organs (Shuai et al., 2002; Bortiri et al., 2006; Koppolu et al., 2013), usually boundary formation is associated with reduced cell division (Wang et al., 2016), so the reduced plant height (**Figure 2E**) and shortened pedicels (**Figures 2B,C**) of pUbi::OsRA2 plants could be explained by the functional conservation of LBD proteins, similar to the stunt phenotype of *AtLOB* ectopic expression in *Arabidopsis* (Shuai et al., 2002).

Maize exhibits two types of reproductive inflorescences, the male tassel and the female ear. Pairs of staminate flowers develop within the tassel while spikelet pairs with two pistillate flowers initiate in the ear (Liu et al., 2009; Thompson and Hake, 2009; Yuan et al., 2009; Zhang and Yuan, 2014). In both maize and barley, *RA2* genes determine the spikelet determinacy and the *ra2* mutant shows increased number of branches in the reproductive organs (Bortiri et al., 2006; Koppolu et al., 2013). However, in the present study, down regulation of *OsRA2* in dsRNAiOsRA2 plants showed distinct pedicel elongation, but not increased branches. Therefore, *OsRA2* does not seem to influence determinacy in rice, which differs from the associations in maize and barley. Although some spikelet pairs of maize *ra2* mutants became single spikelets in the tassel, and some showed longer pedicels, the function of *ra2* in regulating pedicel length is less obvious than *OsRA2* in rice. *RA2* predicts the position of branch anlagen in maize inflorescence (Bortiri et al., 2006); similarly, *OsRA2* functions within rice PB and SB anlagens (**Figures 3C–E**). Therefore, expression profile conservation indicates that *RA2* is critical for shaping the initial steps of inflorescence architecture in both maize and rice.

To date, although a few genes associated with the panicle architecture have been studied, little is known about the underlying mechanism of their functional cross-talks. In the present study, genetic analysis suggested that *LAX1* functions upstream of *OsRA2* in regulating the number of SBs (**Figures 5C,F**), and may also do so in regulating pedicel length (**Figures 5D,E**), although this result might have been influenced by the expression level of *OsRA2* in RNAi plants. The expression of *LAX1* was also influenced by *OsRA2* expression (**Figure 5A**), indicating the possible existence of feed-back regulation between *OsRA2* and *LAX1*. The fact that higher *OsRA2* expression led to an increased number of vascular bundles suggests that it might benefit vascular development. The cross between A989 (gain of function of *RCN2*) and *OsRA2* over expressing lines resulted in plants with more branches and shorter branch lengths, indicating that *OsRA2* acts downstream of *RCN2* in regulating branch lengths, while upstream of *RCN2* in regulating the number of SBs. This provides evidence for the respective regulation of the number and length of branches in the panicle by two parallel pathways.

## Author Contributions

Experimental design: XM and ZS; Experiments: HL, ZD, LL, and JW; Data analysis: JW and ZS; Manuscript preparation: HL, ZD, ZS, and XM; Supervision, funding and reagents: ZS and XM.

## Conflict of Interest Statement

The authors declare that the research was conducted in the absence of any commercial or financial relationships that could be construed as a potential conflict of interest.

## Footnotes

1 http://www.uniprot.org/

2 http://blast.ncbi.nlm.nih.gov/Blast.cgi

3 http://rice.plantbiology.msu.edu/

4 http://blast.ncbi.nlm.nih.gov/Blast.cgi

5 http://www.uniprot.org/

6 http://www.uniprot.org/

7 http://www.dna.affrc.go.jp/PLACE/signalscan.html
