# Supplementary material for: OsRAMOSA2 Shapes Panicle Architecture through Regulating Pedicel Length
Source: Front Plant Sci. 2017 Sep 12;8:1538. doi: 10.3389/fpls.2017.01538 (PMC5601049; doi:10.3389/fpls.2017.01538)
Supplement: Supplementary file 1 [file Presentation_1.PDF]

Title: *OsRAMOSA2* shapes panicle architecture through regulating pedicle length

Huan Lu[1, 2 #], Zhengyan Dai[3 #], Ling Li[1, 3], Jiang Wang[1], Jingliu Zhang[1], Xuexia Miao[3], Zhenying Shi[3*]

Affiliations

[1]National Key Laboratory of Plant Molecular Genetics, Institute of Plant Physiology and Ecology, Shanghai Institutes for Biological Sciences, the Chinese Academy of Sciences, Shanghai, China

[2] University of Chinese Academy of Sciences, Shanghai, China.

[3]Key Laboratory of Insect Developmental and Evolutionary Biology, Institute of Plant Physiology and Ecology, Shanghai Institutes for Biological Sciences, Chinese Academy of Sciences, Shanghai, China

[4]Key Laboratory of Urban Agriculture (South) Ministry of Agriculture, Plant Biotechnology Research Center, School of Agriculture and Biology, Shanghai Jiao Tong University, Shanghai 200240, China

***For correspondence**
Zhenying Shi
zyshi@sibs.ac.cn
Phone: +86-21-54924217, Fax: +86-21-54924015

Running title
A functional study of *OsRA2*

Table S1 The accession numbers of the *RA2* homologs in different species.

RA2 Homologs	Accession Numbers
Schizachyrium sanguineum RA2	I2AVZ2
Loudetia sp.MCE RA2	I2AVY6
Phacelurus digitatus RA2	I2AVZ3
Andropterum stolzii RA2	I2AVZ0
Andropogon hallii RA2	I2AVZ1
Cymbopogon flexuosus RA2	I2AVY8
Chrysopogon gryllus RA2	I2AVY7
Sorghum bicolor RA2	Q27YF1
Hordeum vulgare RA2	Q27YF2
Zea mays RA2	Q27YF3
Oryza sativa RA2	Q9AS62

Table S2 Primer sequences used in this study.

Primers	Sequence(5'-3')	Function
OsRA2-F	CGCAGAAGTTCGCCAACGTC	RT-PCR analysis
OsRA2-R	ATCAAGGCCAAAGCGCAGAT	RT-PCR analysis
RCN1-F	GAACATTGGCATTGAAGTAG	RT-PCR analysis
RCN1-R	CTCTCTGGGCATTGAAGTAG	RT-PCR analysis
RCN2-F	GTTTTCCTATTTCCCCTACAT	RT-PCR analysis
RCN2-R	ATCGGATTACAAAAACAAGTG	RT-PCR analysis
RCN3-F	AGCCCAGCATTGGTATC	RT-PCR analysis
RCN3-R	TTGAAGTAGACAGCAGCGAC	RT-PCR analysis
LAX1-F	CCACGAGCGCGGATCCATCT	RT-PCR analysis
LAX1-R	GCCCATCATCAGCTGCCCCG	RT-PCR analysis
SP1-F	CGGTAACCAAGAGGAAACAAGTG	RT-PCR analysis
SP1-R	CACCACGCACAGTAGCACCTT	RT-PCR analysis
OSH1-F	GCACTGGAAGCCCTCCGACG	*In situ* hybridization analysis
OSH1-R	CATCCATGGCGTGCGTACGTG	*In situ* hybridization analysis
DEP-F	GCTTCAGCTGCCAGTCGTCA	RT-PCR analysis
DEP-R	GCATGGGTTACGACACCGTG	RT-PCR analysis
ACTIN-F	AGGAATGGAAGCTGCGGGTAT	RT-PCR analysis
ACTIN-R	GCAGGAGGACGGCGATAACA	RT-PCR analysis
Q*SP1*-F	CGGTAACCAAGAGGAAACAAGTG	qRT-PCR analysis
Q*SP1*-R	CACCACGCACAGTAGCACCTT	qRT-PCR analysis
XTR1-F	AGCCGTACATCCTGCAGACGA	qRT-PCR analysis
XTR1-R	GCCCAGGTCCTTGCTGTTCT	qRT-PCR analysis
Q*OsRA2*-F	GTCCTACCTTACGCTTCC	qRT-PCR analysis
Q*OsRA2*-R	TCTCTGATCCTGAGTTGG	qRT-PCR analysis
Q*LAX1*-F	CATCAGATGATGCAGCAAGC	qRT-PCR analysis
Q*LAX1*-R	AGACACAGCAAGGCAAAGGA	qRT-PCR analysis
QActin-F	CAGCCACACTGTCCCCATCTA	qRT-PCR analysis
QActin-R	GCAGGAGGACGGCGATAACA	qRT-PCR analysis
*OsRA2-Bam*HIA	GGGATCCAAATGGCATCCTCGTCGAGCACC	pUbi::OsRA2 plasmid construction
*OsRA2-Kpn*I	GGGTACCATCAAGGCCAAAGCGCAGAT	pUbi::OsRA2 plasmid construction
OsRA2-RNAiF	GGGAGCTCGGATCCGAAATGGCATCCTCGTCGAGCACC	pdsRNAiOsRA2 plasmid construction
OsRA2-RNAiR	GGGACTAGTGGTACCGCGGGAAGTAAGGAGCGAACACG	pdsRNAiOsRA2 plasmid construction
*OsRA2-Bam*HIB	GGGGATCCATGGCATCCTCGTCGAGCACC	OsRA2-GFP plasmid construction
*OsRA2-Pst*I	GGGCTGCAGCATGCTGCTGTCTCCTCCTTCC	OsRA2-GFP plasmid construction
OsRA2-insitu F	GGGAATTCATCAAGGCCAAAGCGCAGAT	*In situ* hybridization analysis
OsRA2-insitu R	GGGATCCCGCAGAAGTTCGCCAACGTC	*In situ* hybridization analysis
Ubi90+	GCCCTGCCTTCATACGCTATT	Examination of the dsRNAiOsRA2 transformants
ocs-160	CGATAGTAACGGGTGATATA	Examination of the dsRNAiOsRA2 transformants
Osa_yRA2EcoRI	GGGAATTCATGGCATCCTCGAGCACC	pEG202-OsRAMOSA2 plasmid construction
Osa_yRA2BamHI	GGGGATCCTCACATGCTGCTGTCTCCTCCTTCC	pEG202-OsRAMOSA2 plasmid construction

Figure legends

Figure S1 Expression analysis of *OsRA2* in transgenic plants.

(A) Semi-quantitative RT-PCR analysis of *OsRA2* in T0 generation dsRNAiOsRA2 and pUbi::OsRA2 transgenic plants. The *actin* was used as an internal control. (B) qRT-PCR analysis of *OsRA2* in T2 generation dsRNAiOsRA2 and pUbi::OsRA2 transgenic plants. Student's *t*-test at *P<0.05 and **P<0.01 compared with WT (ZH11).

Figure S2 (A-D) Statistical analysis of panicle traits among ZH11, dsRNAiOsRA2 and pUbi::OsRA2 plants. Values in A-D are means ± SE. n=30 panicles in A-D.

(E) Statistical analysis of heights of ZH11, dsRNAiOsRA2, and pUbi::OsRA2 plants.

Figure S3 Measurement of pedicle thickness in T2 generation dsRNAiOsRA2 and pUbi::OsRA2 transgenic plants.

Student's *t*-test at *P<0.05 and **P<0.01 as compared with WT (ZH11).

Figure S4 Phenotype of p35S::GFP-OsRA2 transgenic plants.

(A) Height of p35S::GFP-OsRA2 transgenic plants. (B) Panicle phenotype of p35S::GFP-OsRA2 transgenic plants.

Figure S5 Semi-quantitative RT-PCR analysis of panicle-related genes in dsRNAiOsRA2 and pUbi::OsRA2 transgenic plants.
