# Supplementary figures and images for: OsRAMOSA2 Shapes Panicle Architecture through Regulating Pedicel Length
Source: Front Plant Sci. 2017 Sep 12;8:1538. doi: 10.3389/fpls.2017.01538 (PMC5601049; doi:10.3389/fpls.2017.01538)

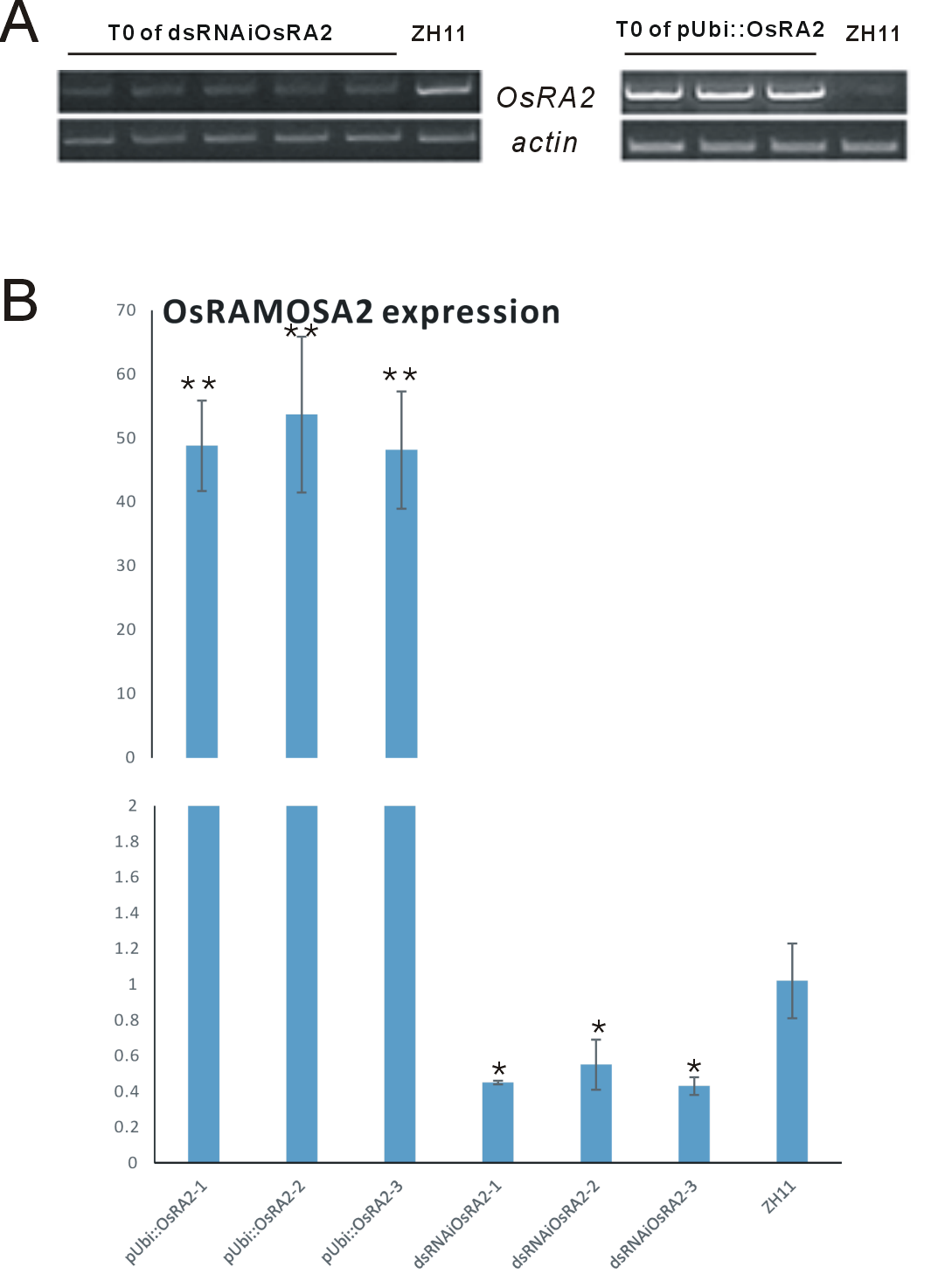

Supplement: Supplementary file 2 [file Image_1.TIF]

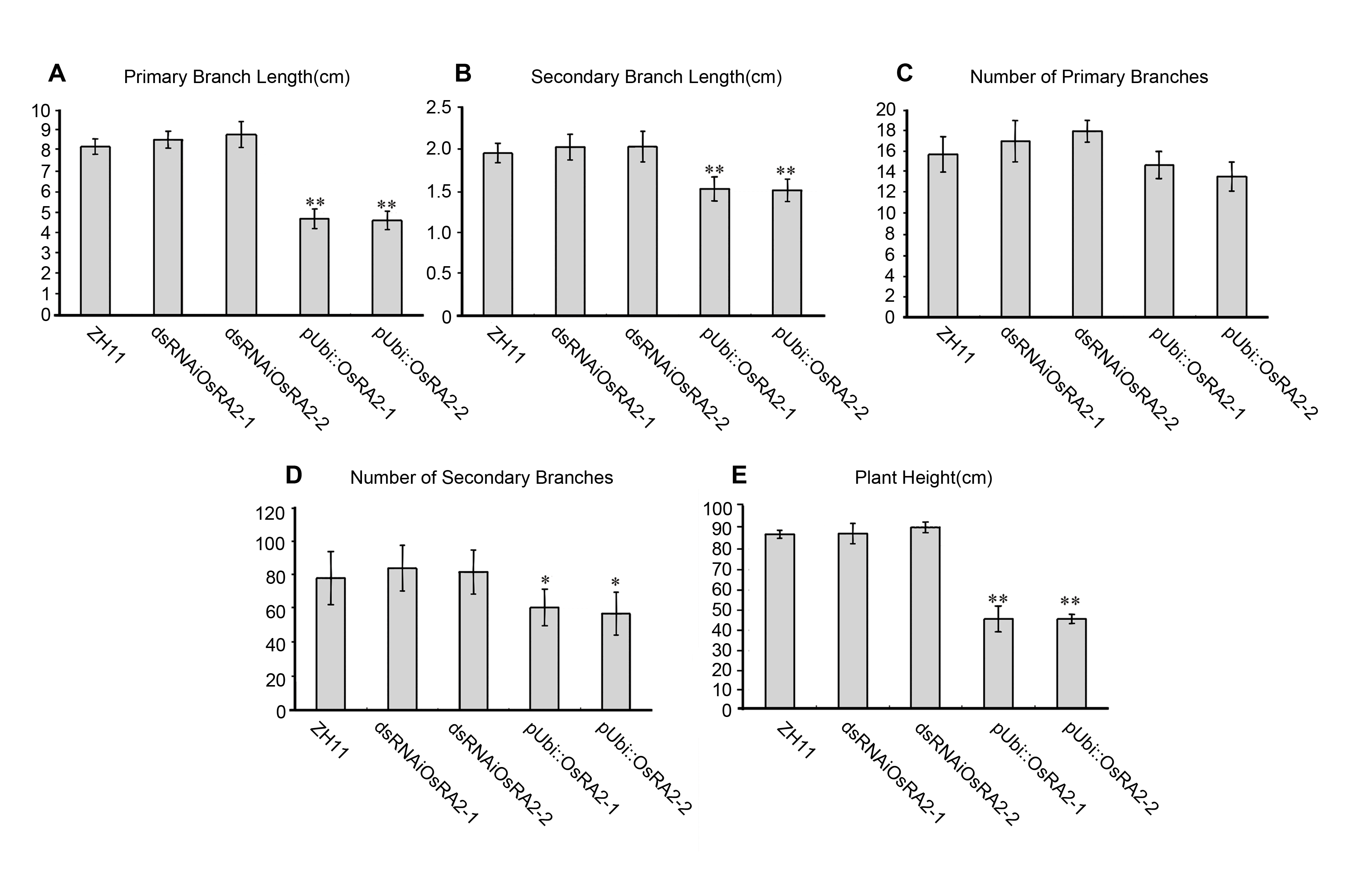

Supplement: Supplementary file 3 [file Image_2.TIF]

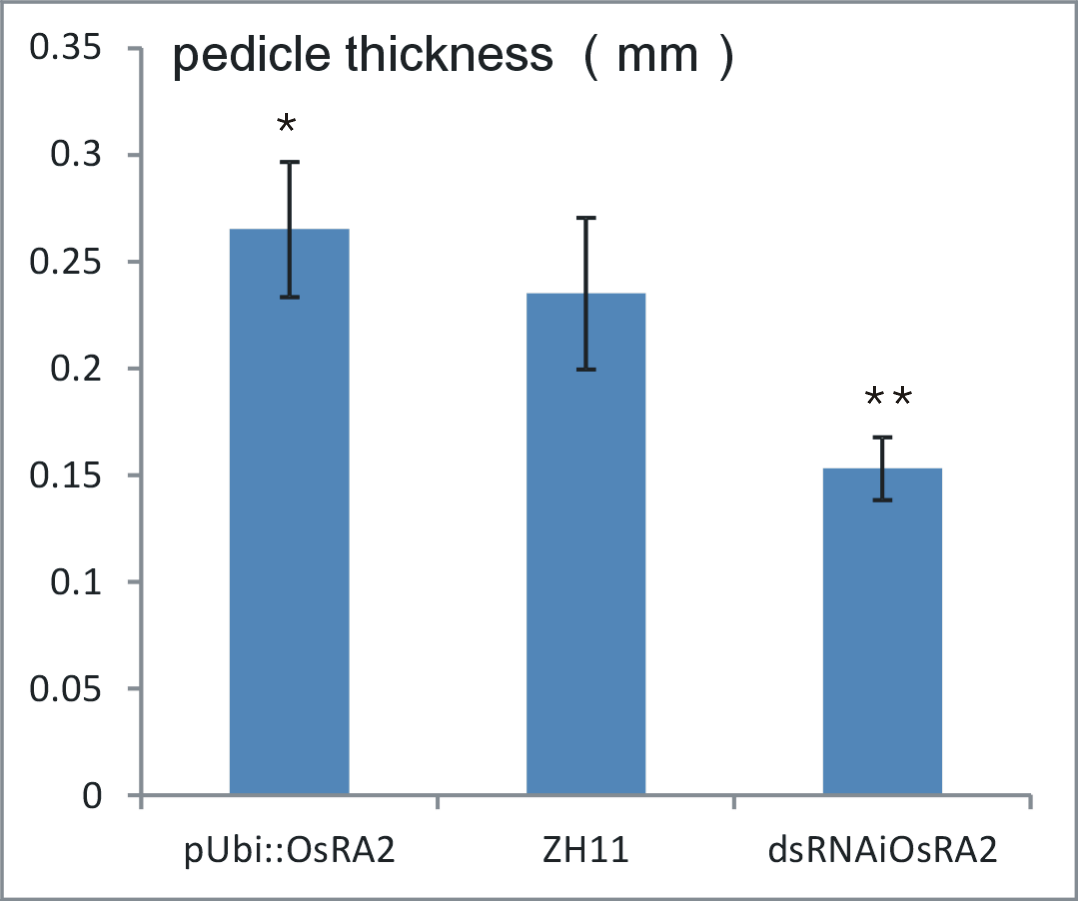

Supplement: Supplementary file 4 [file Image_3.TIF]

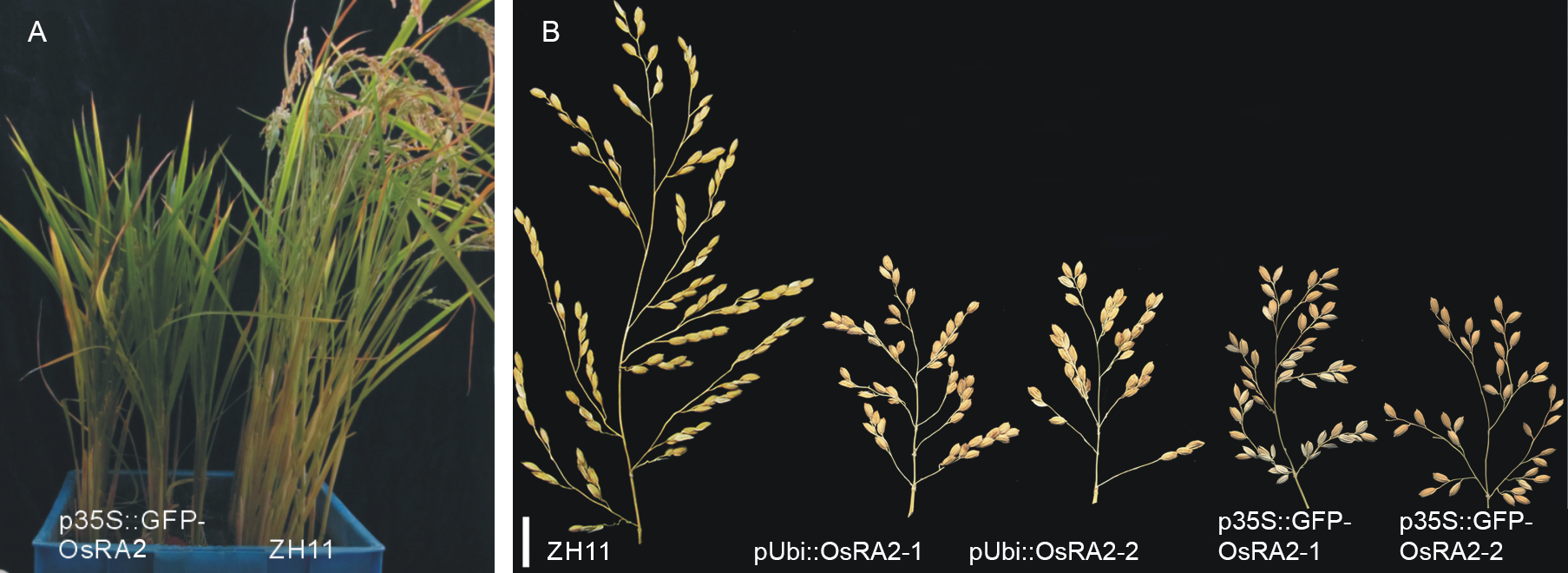

Supplement: Supplementary file 5 [file Image_4.TIF]

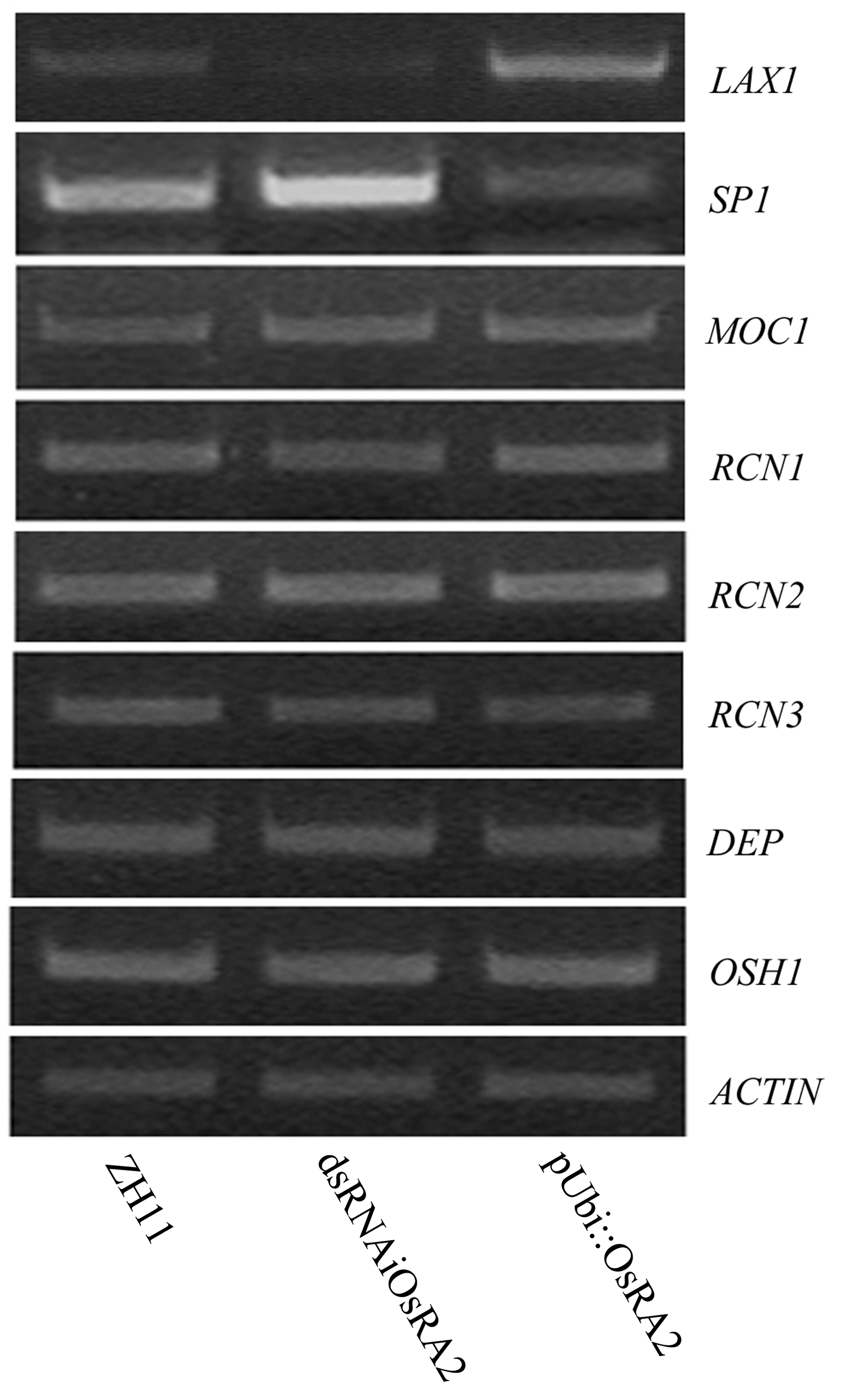

Supplement: Supplementary file 6 [file Image_5.TIF]
